# Effects of physical activity and exercise on the cognitive function of patients with Alzheimer disease: a meta-analysis

**DOI:** 10.1186/s12877-019-1175-2

**Published:** 2019-07-02

**Authors:** Rui-xia Jia, Jing-hong Liang, Yong Xu, Ying-quan Wang

**Affiliations:** Department of Social Medicine, Jiangsu Key Laboratory of Preventive and Translational Medicine for Geriatric Diseases, School of Public Health, Medical College of Soochow University, No.199 Ren Ai Road, Suzhou, People’s Republic of China 215123

**Keywords:** Physical activity, Exercise, Alzheimer’s disease, cognition, Meta-analysis, Elderly, Older adults

## Abstract

**Background:**

Alzheimer’s disease (AD), as the most common cause of dementia, brings huge economic burden for patients and social health care systems, which motivates researchers to study multiple protective factors, among which physical activity and exercise have been proven to be both effective and economically feasible.

**Methods:**

A systematic literature search was performed for eligible studies published up to November 1st 2018 on three international databases (PubMed, Cochrane Library, and Embase) and two Chinese databases (Wanfang Data, China National Knowledge Infrastructure). All analyses were conducted using Stata 14.0. Due to heterogeneity between studies, a random-effects model was used for this meta-analysis. Meta-analysis was used to explore if physical activity and exercise can exert positive effects on cognition of elderly with AD and subgroup analyses were conducted to find out if there are dose-response effects.

**Results:**

A total of 13 randomized controlled trials were included with a sample size of 673 subjects diagnosed with AD. Intervention groups showed a statistically significant improvement in cognition of included subjects measured by the MMSE score (SMD = 1.12 CI:0.66~1.59) compared to the control groups. Subgroup analyses showed different amounts of physical activity and exercise can generate different effects.

**Conclusions:**

As one of few meta-analyses comparing different quantities of physical activity and exercise interventions for AD in details, our study suggests that physical activity and exercise can improve cognition of older adults with AD. While the concomitant effects on cognition functions of high frequency interventions was not greater than that of low frequency interventions, the threshold remains to be settled. However, more RCTs with rigorous study design are needed to support our findings.

**Electronic supplementary material:**

The online version of this article (10.1186/s12877-019-1175-2) contains supplementary material, which is available to authorized users.

## Background

According to World Alzheimer Report 2016, about 47 million people struggled with dementia worldwide. This number was predicted to increase to more than 131 million by 2050, as the size and proportion of population age 65 and older continue to escalate across the world. There is also huge health care cost caused by dementia. The total estimated cost of dementia was US$818 billion worldwide, which is set to rise as the number of people with dementia increases [1]. There are mainly 8 causes of dementia including Alzheimer’s disease(AD), Vascular dementia, Dementia with Lewy bodies, Mixed dementia, Frontotemporal lobar degeneration, Parkinson’s disease dementia, Creutzfeldt-Jakob disease and Normal pressure hydrocephalus, among which AD is the most common cause of dementia and accounts for about 60 to 80% of cases [2].

### What is AD: definition, symptoms and risk factors

AD is a chronic neurodegenerative disorder starting and developing insidiously, whose typical primary groups of symptoms are: (1) cognitive dysfunction; (2) psychiatric symptoms and behavioral disturbances; (3) difficulties with performing activities of daily living. These symptoms progress from mild memory loss to very severe dementia [3]. With the disease deteriorating, patients would lose their bodily functions and life finally. Studies show that the typical life expectancy following diagnosis is three to nine years despite of various speed of progression on different individuals [4, 5]. AD is a devastating disease both expensive in economy and painful in emotion for patients and their families, yet few final conclusion has yet been reached on the precise biological changes that cause AD, why its progressions vary among different patients, and how to prevent, slow or stop it [2].

The heavy damage to the society, patients and their families caused by AD motivates researchers in different fields to study risk factors and possible ways to conduct prevention and intervention against AD. For example, Miia Kivipelto et al [6] reported advanced age, family history, apolipoprotein E (apoE) ε4 allele, physical inactivity, high dietary fat intake, alcohol drinking and smoking were risk factors for AD. Cedazo-Minguez A [7] had similar opinion that risk factors for AD included advanced age, family history of dementia, low educational accomplishment and presence of the ε4 isoform of the apolipoprotein E (apoE). Rolland Y et al [8] hold that modifiable lifestyle factors such as inactivity may affect the development and progression of AD. Fratiglioni L et al [9] reported that three lifestyle factors can slow the rate of cognitive decline and preventing dementia: a socially integrated network, cognitive leisure activity, and regular physical activity. There are studies suggesting physical activity has the most support as protective against the deleterious effects of age on health and cognition [10, 11]. Since physical activity and exercise are appealing low-cost and low-risk alternative treatment [8], more and more trails have been conducted to examine the effects of physical activity and exercise on the cognition of patients with AD.

### Effects of physical activity and exercise

Recently, results from randomized controlled trails (RCTs) have suggested that people should adopt physical activity and exercise to alleviate the negative impact of aging on their cognitive function. A randomized trail conducted by Lautenschlage NT [12] in 2008 showed physical activity and exercise may slow down cognitive decline, which is in agreement with Kramer AF [13]. Heyn P et al [14] reported physical activity and exercise had positive effects on cognition among those with cognitive decline in a meta-analysis.

Evidence from all kinds of trails suggests that physical activity and exercise can to some extent improve cognition performance among patients with cognitive impairment, yet it is still unclear which combinations of frequency, intensity, time, and type of exercise can exert a better effect on improving cognition of older adults diagnosed with AD.

To find the optimal intervention way, it is urgent to answer how to conduct physical activity and exercise RCTs to improve cognition in terms of the amount of physical activity and exercise. This meta-analysis was conducted to study the dose-response effects of physical activity on cognition of patients with AD as a need-to-be settled question. The findings should provide bases for establishing guidelines and recommendations for future physical activity and exercise interventions for older adults with AD.

## Methods

### Search strategy

A systematic literature search was performed for eligible studies published before November 1st 2018 on three international databases (PubMed, Cochrane Library, and Embase) and two Chinese databases (Wanfang Data, China National Knowledge Infrastructure). Key words as “exercise” OR “physical activity” OR “physical activities” OR “physical training” AND “Alzheimer Disease” OR “Alzheimer’s Disease” OR “Alzheimer-Type Dementia” were searched. 5450 studies were retrieved in this search, which were supplemented by manual searches through reference lists of published reports. Titles and abstracts from a final total of 5462 studies were then reviewed for further inclusion. All analyses were based on published studies, therefore no ethical approval and patient consent are required.

### Criteria for inclusion

**Fig. 1:**
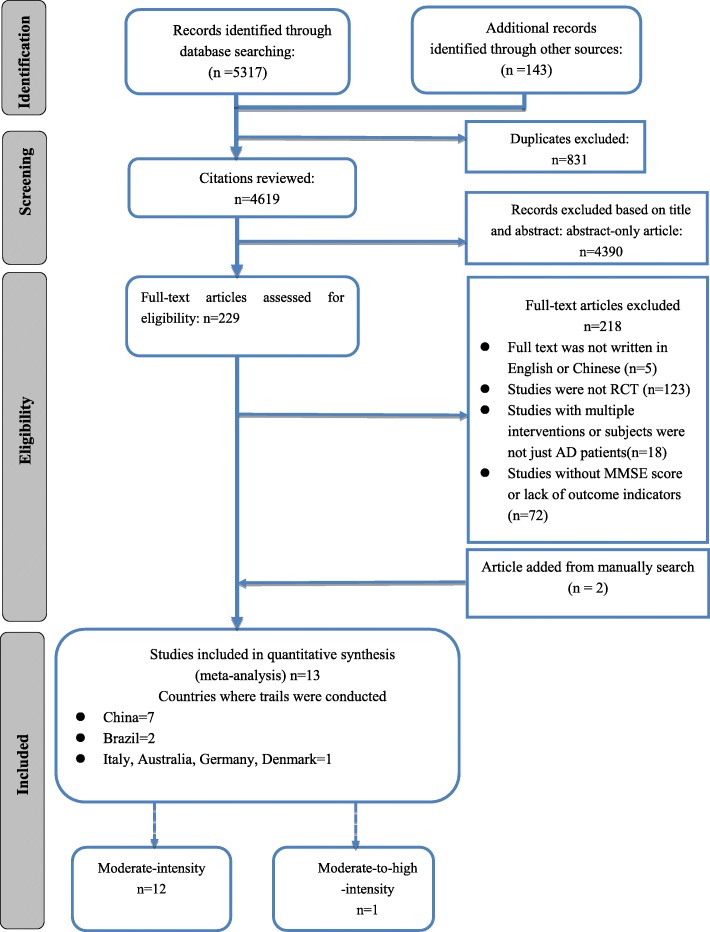
Literature review flowchart. (AD, Alzheimer’s disease; MMSE, Mini-Mental State Examination; RCT, Randomized controlled trial)

Studies were included that met the following inclusion criteria:(1) included subjects diagnosed with AD; (2) involved an exercise-only intervention in the experimental group under the guarantee of basically medical care; (3) included a non-diet, non-exercise control group under the guarantee of basically medical care; (4) pre- and post-intervention cognitive function measurements were reported with means, standard deviations (SDs) of Mini-Mental State Examination(MMSE) for both exercise and control groups; (5) written in English or Chinese. Two authors (Jiao Ruixia and Liang Jinghong) independently reviewed full texts of all articles that were considered relevant for inclusion in this review. A third author was consulted in cases of disagreements between these two authors. The study selection process is described in Fig. 1.

### Criteria for exclusion

Studies that clearly did not meet initial criteria were rejected on initial review. Reviews, conference papers, abstracts without available full text and studies written in languages other than English and Chinese were also excluded. Studies that were qualitative or investigated the effects of exercise on cognitive function in combination with other interventions, such as cognition therapy or cognitive stimulation, were excluded.

### Data extraction

Titles and abstracts of studies identified in the initial search were imported into EndNote for Preliminary screening. After duplicates, studies that are clearly irrelevant to the effects of physical activity and exercise on AD and animal trails were removed; full texts of potentially relevant papers were imported for further screening. Two authors (RXJ, JHL) performed an initial screening of titles and abstracts independently followed by a screening potential relevant full texts guided by inclusion criteria. A third reviewer (YQW) was consulted for any screening disagreement.

From each study, we collected the following data if available: year of publication, sample size and participants’ characteristics (age, number of participants from experimental groups and control group, gender proportion, country, pre- and post-intervention MMSE score, adverse effects and conclusions), intervention characteristics (type, length of whole duration, frequency, time of single session, intensity of intervention). Relevant information was extracted by two reviewers independently onto a standard template.

### Risk of bias

Studies meeting inclusion criteria were individually scored by two authors (RXJ, JHL) independently according to the Cochrane risk of bias tool, the third author (YQW) would be consulted when disagreement appeared. 7 items regarding risk of bias was assessed: random sequence generation, allocation concealment, blinding of participants and personnel, blinding of outcome assessment, incomplete outcome data, selective outcome reporting, and other sources of bias. The included RCTs were classified as being at low risk, high risk, or unclear risk in the above fields.

### Data analysis

All analyses were conducted using Stata 14.0. Due to heterogeneity between studies, a random-effects model was used for this meta-analysis. Subgroup analyses were conducted to explore the differences among the effects of various types of physical activity and exercise on cognitive performance of subjects with AD.

#### Meta-analysis

Random-effects meta-analysis was selected due to the existence of methodological heterogeneity across studies. Standardized mean differences (SMD) were pooled because outcomes were continuous. Heterogeneity across studies was quantified using I squared statistic (variation in SMD attributable to heterogeneity).

#### Subgroup analysis

Studies were grouped based on country, time of a single session, intervention time per week, frequency per week and length of whole duration. Intervention effects were estimated within subgroups and compared across subgroups to identify components that modify the intervention effects.

## Results

### Study selection

The original search identified 5462 citations. 229 citations underwent a full-text screen by two independent reviewers (RXJ, JHL), after duplicates, studies irrelevant to AD and exercise or physical activity, animal trails and reviews were removed. A total of 13 trials were included in the final review (see Additional file 1. A full report of the selection process can be found in the PRISMA diagram in Fig. 1.

### Description of studies

Descriptive characteristics for 13 included studies are presented in Table 1. Of the 13 trials, seven were based in China [15–21], two in Brazil [22, 23], and one each in Italy [24], Australia [25], Germany [26] and Denmark [27]. In total, the trials included 673 participants, with 200 recruited from one trial [27]. Four studies reported 26 drop-out subjects in total, which leaved 647 subjects. The 13 included studies were published between 2008 and 2017. All studies reported beneficial effects of physical activity on cognition of patients with AD.

**Table 1:** Baseline characteristics and intervention details of included studies

Author	Year	Country	Sample size		Age (Mean±SD)		Intervention type	Minutes per session	Hours per week	Days per week	Duration	Intensity
			IG	IG	IG	CG						
Laís Fajersztajn	2008	Brazil	5	5	78.40 (6.43)	76.40 (7.50)	function-task physical activity	60	1	1	12	moderate
Massimo Venturelli	2011	Italy	12	12	83.00 (6.00)	85.00 (5.00)	exercise (walking)	30	2	4	24	moderate
Anthea Vreugdenhil	2012	Australia	20	20	73.50 (-----)	74.70 (-----)	daily exercises and walking	30	2.2	7	16	moderate
Cynthia Arcoverde	2013	Brazil	10	10	78.50 (-----)	79.00 (-----)	treadmill walking	30	1	2	12	moderate
Wang Ying	2014	China	13	26	71.60 (5.80)	70.60 (8.40)	daily exercises and walking	30	1.5	3	12	moderate
Wang Shiyan	2014	China	24	24	70.29 (7.25)	71.10 (8.16)	cycle ergometer exercise	40	2	3	12	moderate
Wang Wei	2014	China	30	30	71.19 (7.04)	70.04 (8.09)	aerobic exercise	40	2	3	24	moderate
Kunze M	2015	Germany	15	15	72.40 (4.34)	70.67 (5.41)	physical activity	30	1.5	3	12	moderate
Kristine Hoffmann	2015	Denmark	107	93	69.80 (7.40)	71.30 (7.30)	aerobic exercise	60	3	3	16	moderate to high
Si-YuYang	2015	China	25	15	72.00 (6.69)	71.92 (7.28)	aerobic exercise	40	2	3	12	moderate
Yan Lanyun	2015	China	18	18	72.10 (6.10)	70.60 (6.30)	aerobic exercise	30	1	2	24	moderate
Mu Haiyan	2016	China	39	39	72.90 (5.36)	73.69 (4.56)	brisk walking	60	3	3	16	moderate
Liu Yin	2017	China	24	24	70.90 (9.20)	70.30 (7.70)	aerobic exercise	40	2	3	12	moderate

*SD* standard deviation, *IG* intervention group, *CG* control group. ----- data not available

**Fig. 2:**
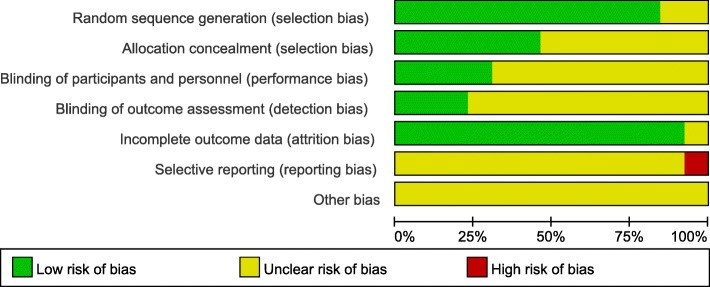
Assessment for risk of bias

**Fig. 3:**
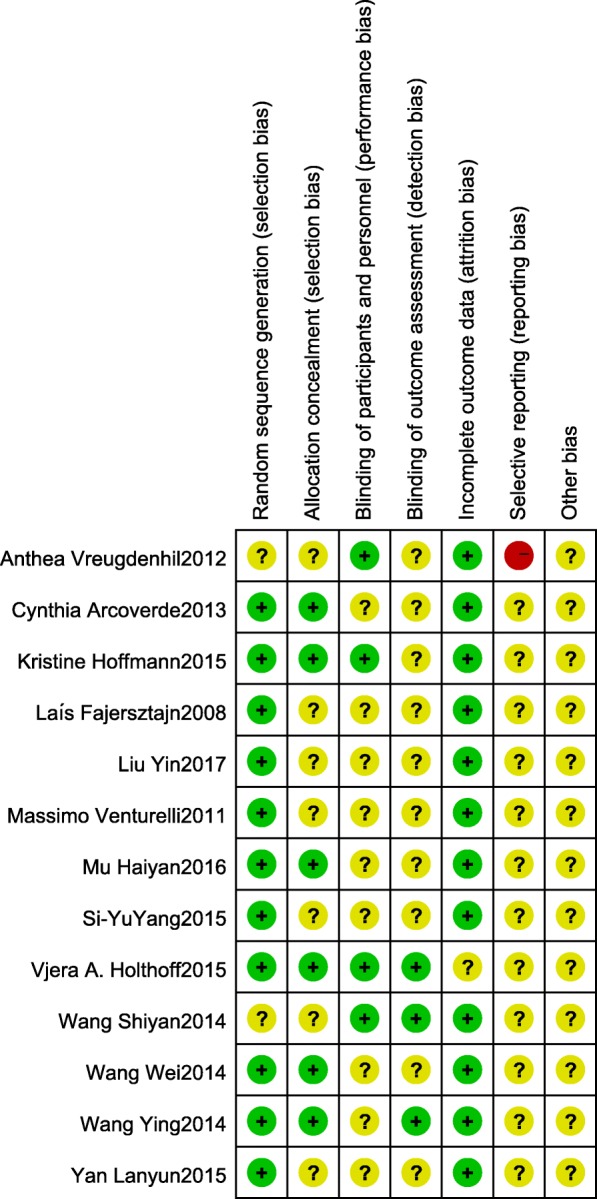
Assessment for the risk of bias in included studies

Among all included studies, random assignment procedure were reported in 7 studies; Only 5 studies reported a blinding procedure; Analyses accounting for patient drop-out during intervention were presented in 5 studies; All studies described the exercise session duration and reported MMSE scores. Figures 2 and 3 presented the degree of risk of bias for all studies included.

### Participants’ characteristics

All included trials contained a sample size of 673 subjects diagnosed with AD. Exercise and control groups were composed of 342 and 331 subjects respectively. A total of 26 subjects dropped out, among which 13 were from experimental groups and the other 13 were from control groups, leaving 647 subjects completing these RCTs. The percentage of female participants was 56.16% at baseline. Subjects’ average age was 73.53 (range from 51 to 89) with a mean Mini Mental State Examination score of subjects was 20.09 at baseline (Table 1).

### Intervention characteristics

Table 1 showed that of 13 included studies, only one carried out moderate-to-high intensity intervention, the rest of included studies conducted moderate intensity exercise or physical activity. Aerobic exercise was chosen by 11 studies. All interventions were conducted at an average of 40 min a session ranging from 30 to 60 min. Interventions were conducted at least one hour a week. Length of whole duration ranges from 12 to 24 weeks, mean intervention duration was 16.92 weeks. Only one study reported follow-up MMSE score 3 months after the intervention. One study reported adverse effect and one study did intention-to-treat (ITT) analysis.

### Meta-analysis

#### Primary outcome

**Fig. 4:**
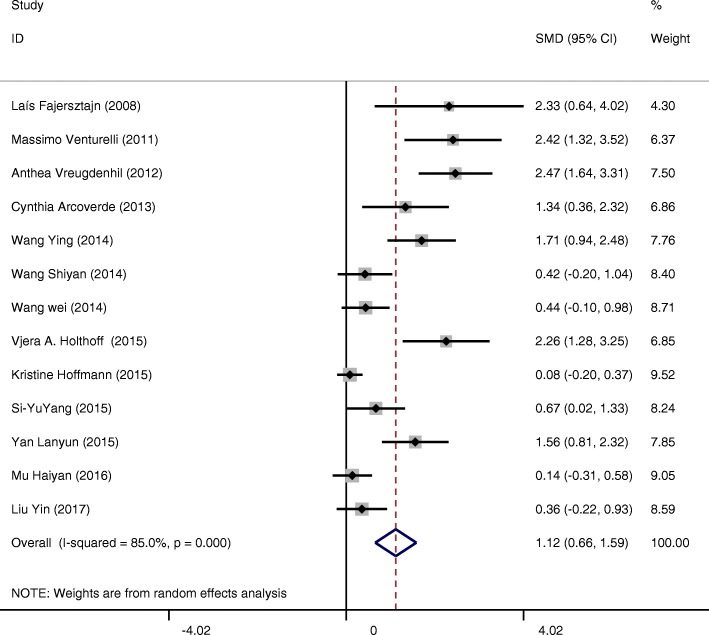
Effects of physical activity and exercise on the outcome of cognitive function of patients with AD (13 studies)

**Fig. 5:**
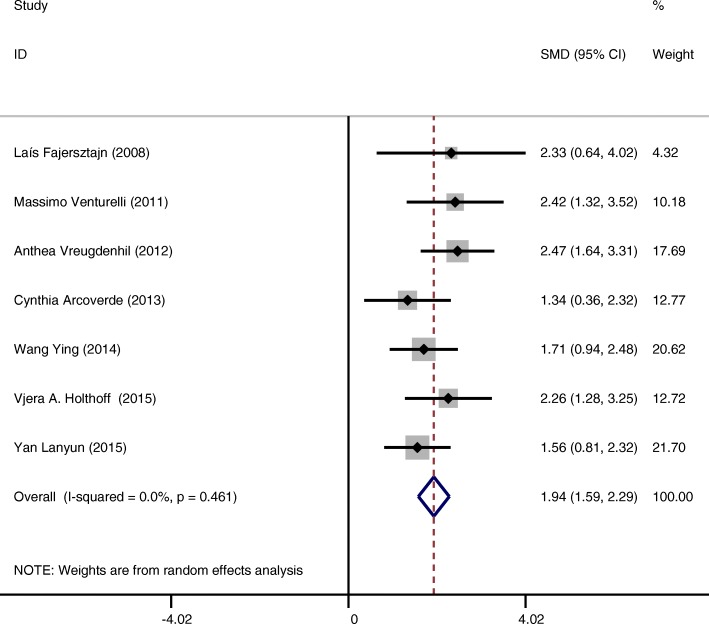
Effects of physical activity and exercise on the outcome of cognitive function of patients with AD (7 studies)

Intervention groups showed a statistically significant improvement in cognition of included subjects measured by the MMSE score (SMD = 1.12 CI:0.66~1.59 I-squared = 85.0% *p* = 0.000) compared to the control groups (Fig. 4). After exclusion of 6 studies [16–18, 20, 21, 27] based on funnel plots, we found positive overall random effects of physical activity or exercise interventions on cognitive function (SMD = 1.94 CI:1.59~2.29 I-squared = 0.0% *p* = 0.461) (Fig. 5). To explain the heterogeneity between 13 studies and find modifiable factors of physical activity and exercise, we did five further subgroup analyses.

#### Secondary outcome

##### Subgroup analysis

**Fig. 6:**
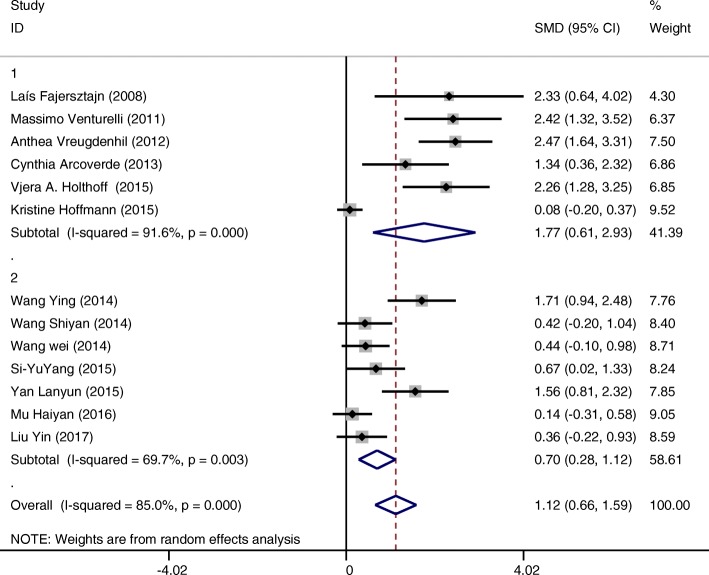
Subgroup analysis on the outcome of cognitive function categorized by country (Group 1 interventions in countries other than China; Group 2 interventions in China)

**Fig. 7:**
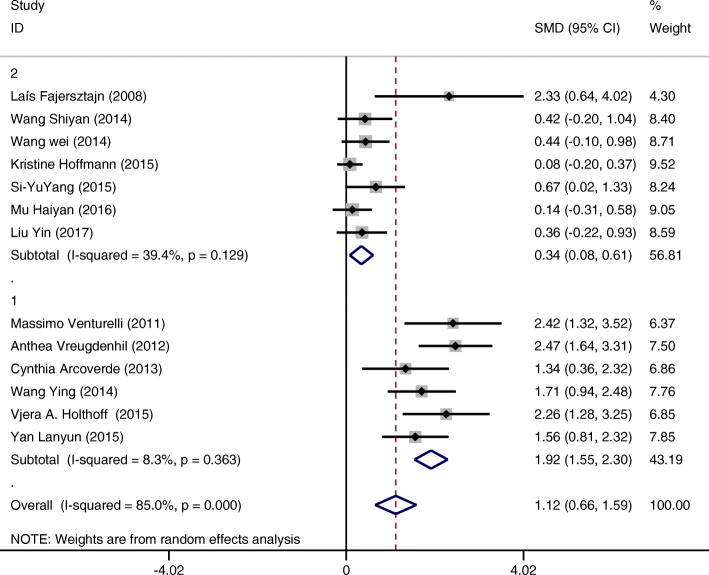
Subgroup analysis on the outcome of cognitive function categorized by minutes of intervention per session (Group 1 up to 30 min per session; Group 2 more than 30 min per session)

- 1st subgroup categorized by country
  Group 1 (interventions conducted in countries other than China) (SMD = 1.77 CI:0.61~2.93 I-squared = 91.6%, *p* = 0.000) showed a greater positive effect on cognition compared to group 2 (interventions conducted in China) (SMD = 0.70 CI:0.28~1.12 I-squared = 69.7%, *p* = 0.000). (Fig. 6). Although it is hard to draw a final conclusion on this due to weak evidence, the tendency is noteworthy.
- 2nd subgroup categorized by minutes of intervention per session
- Group 1 (interventions conducted up to 30 min per time) (SMD = 1.92 CI:1.55~2.30 I-squared = 8.3%, *p* = 0.363) showed greater effectiveness for improving cognition of patients compared to group 2 (those conducted more than 30 min per time) (SMD = 0.34 CI:0.08~0.61 I-squared = 39.4%, *p* = 0.129) (Fig. 7).

#### 3rd subgroup categorized by hours of intervention per week

**Fig. 8:**
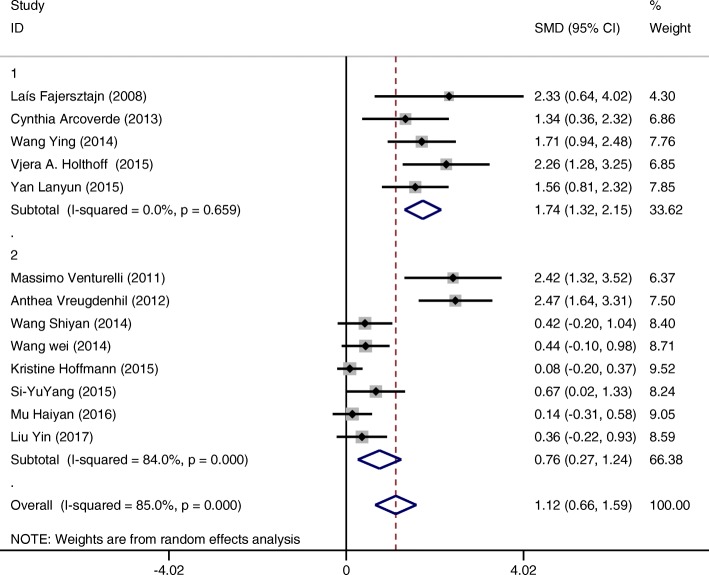
Subgroup analysis on the outcome of cognitive function categorized by hours of intervention per week (Group 1 up to two hours per week; Group 2 more than 2 h per week)

Interventions conducted up to 2 h per week(SMD = 1.74 CI:1.32~2.15 I-squared = 0.0%, *p* = 0.659) have a tendency to show greater effectiveness for improving cognition of patients compared to those conducted more than 2 h per week (SMD = 0.76 CI:0.27~1.24 I-squared = 84.0%, *p* = 0.000) (Fig. 8).

#### 4th subgroup categorized by frequency of intervention per week

**Fig. 9:**
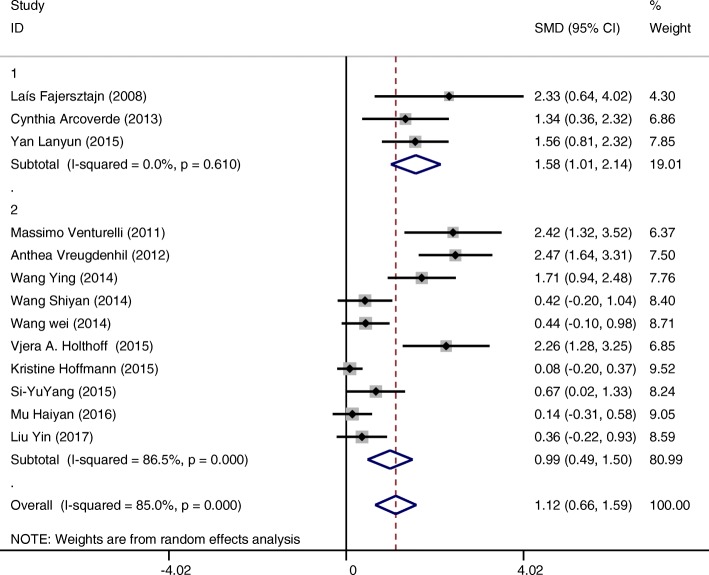
Subgroup analysis on the outcome of cognitive function categorized by frequency of intervention per week (Group 1 up to three times per week; Group 2 more than 3 times per week)

Interventions conducted less than 3 times including 3 times per week(SMD = 1.58 CI:1.01~2.14 I-squared = 0.0%, *p* = 0.61) showed greater effect on improving cognition of patients compared to those conducted more than 3 times per week (SMD = 0.99 CI:0.49~1.50 I-squared = 86.5%, *p* = 0.000) (Fig. 9).

#### 5th subgroup categorized by length of whole intervention duration

**Fig. 10:**
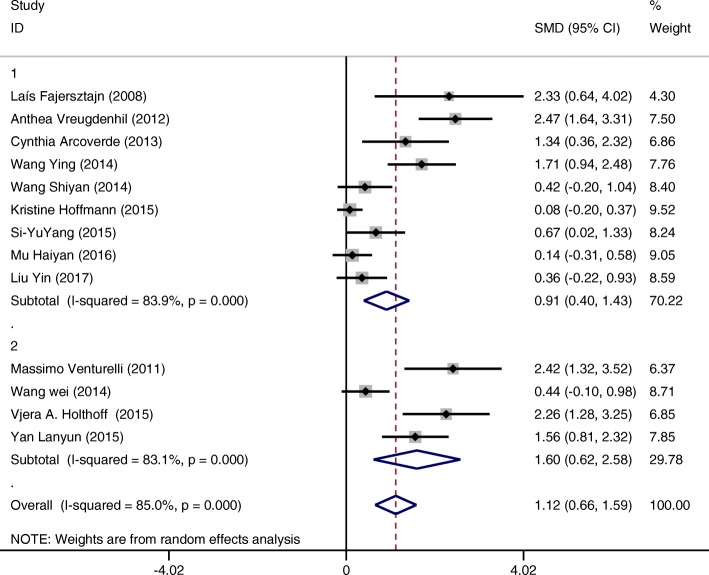
Subgroup analysis on the outcome of cognitive function categorized by length of whole intervention duration (Group 1 duration up to 16 weeks; Group 2 duration more than 16 weeks)

Interventions conducted more than 16 weeks (SMD = 1.60 CI:0.62~2.59 I-squared = 83.1%, *p* = 0.000) showed greater effect on improving cognition of patients compared to those conducted up to 16 weeks. (SMD = 0.91 CI:0.40~1.43 I-squared = 83.9%, *p* = 0.000) (Fig. 10).

#### Publication bias

No obvious publication bias was found.

## Discussion

There are a growing number of studies suggesting that physical activity and exercise have positive effects on cognitive function of the elderly [28–30]. A systematic review [31] showed the relation between exercise and improvement in overall cognitive function as seen by an improvement in scores on the MMSE and the ADAS–Cog. A previous meta-analysis of 29 randomized controlled trials (*n* = 2049) showed that aerobic exercisers exerted positive effects on executive function [32]. Sergio Machado et al [33] suggested that physical exercise was an important neuroprotective modulator, controlling the disease and amplifying brain functions in a significant way. The findings of a review [34] confirmed that in most cases physical activities had a positive effect on the improvement of cognitive decline in AD. In a systematic review, Ruth Stephen et al [35] hold that physical activity is particularly protective against AD. A new review [36] concluded that physical exercise determines positive biological and psychological effects that affect the brain and the cognitive functioning. Tyndall, A. V. et al [37] proposed physical activity, particularly aerobic exercise, through a number of mechanisms has positive effects on blood biomarkers, physiology, and psychological factors associated with cognitive functioning. The positive effect of physical activity and exercise on cognition has been proved by aforementioned studies, which is in consistent with our findings. All improvement in scores on the MMSE from intervention groups of included studies showed that patients in intervention groups with a period of physical activity or exercise had an improved cognition performance while those in control groups did not. But it is noteworthy that there is subtle difference between various trails conducted in China and other countries, for which the reason is left to be discovered. In this term, there should be more RCTs involving subjects from different political, cultural, geographic, religious and dietary backgrounds to explore if there are any cofactors in these above respects affecting the cognition of elderly with AD together with physical activity and exercise.

Even though there is robust evidence supporting the effects of physical activity and exercise on cognition of patients with AD, the mechanism is still left to be discovered. The mechanisms Patrick J. Gallaway et al. [34] proposed several potential mechanisms as physical activity can increase blood flow to the brain, improve sleep quality, improve cardiovascular and metabolic health, prevent and treat depression. However, the potential confounding effects of the aged brain cognitive reserve can not be neglected. Francesca Gelfo [38] et al. described animal studies focusing on neuroanatomical and molecular effects of environmental enrichment to address the biological bases of the experience-dependent “brain reserve”, which is to say that enriched environment is beneficial for brain cognitive reserve. A population-based study [39] reported that environments that offer physical, cognitive or social interaction stimuli may be conductive in increasing cognitive reserve or compensate for damaging processes. A study conducted in German [40] showed that increased left frontal cortex-connectivity was associated with higher reserve in the memory domain. Nicolai Franzmeier et al [41] then reinforced this theory by proposing that interactions between the left frontal cortex and specific functional networks supported reserve in AD. In a review article [42], the mechanisms underlying the neuroprotective effect of brain reserve was also discussed. Based on these studies, in addition to the benefit of physical activity and exercise on cognition, it should also be considered that the contribution brought by brain reserve to improve the cognition performance of patients with AD. Hence future studies on the effects of physical activity and exercise need to consider the confounding effect of brain reserve to find out to what exact extent physical activity and exercise can improve cognition performance of patients with AD.

It is well established that physical activity and exercise are beneficial for AD patients’ cognition, yet few studies proposed a specific way of exercise or physical activity that could exert a positive effect on AD. Öhman H [43] et al. found the outcomes of cognitive tests did not correlate with the number of sessions attended. Shane P. Cass [44] et al. stated that it is not clear if the relationship is dose dependent. Yet on the contrary Yu Fang stated that different ways of exercise or physical activity may confer different effects [45]. Roach K E et al. concluded that specificity of training is one important training parameter in designing successful exercise programs [46]. BM Brown [47] held that significant cognitive benefits required a threshold level of exercise or physical activity. Our study indicated that adopting appropriate exercise or physical activity is of vital importance to improve cognition of patients with AD.

There has also been a lot of controversy on the dose-response relationship between physical activity or exercise and cognition. Several institutions and studies recommended different versions of quantity and quality of exercise for preventing cognition decline.

The American College of Sports Medicine and the American Heart Association [48] suggest that to promote and maintain health, older adults need moderate-intensity aerobic physical activity for a minimum of 30 min on five days each week or vigorous-intensity aerobic activity for a minimum of 20 min on three days each week. According to the World Health Organization [49], people over 65 years old fully functional in cognition should adopt a weekly minimum of 150 min of moderate-intensity aerobic or 75 min of vigorous-intensity aerobic activity with additional muscle-strengthening exercises to reduce the risk of cognitive decline, which also suits individuals with neurodegenerative disease. Neal D. Barnard et al. [50] state that adopting 40 min of brisk walking 3 times per week as a routine activity can improve cognitive function, however in our study 30 min physical activity or exercise appears to be more effective. Although with the limitation of comparison among different types of physical activity and exercise, we could not conclude on which combination is best, but from the whole picture we could see trails conducted 30 min do have a better effects on cognition function compared to those conducted more than 30 min. Lucia A [51] et al. hold that it might be possible to prevent the risk of cognitive deterioration by promoting ≥30 min on five days a week of moderate-intensity aerobic PA, or vigorous-intensity aerobic PA for a minimum of 20 min on three days each week.

After comparing 12 trials conducting moderate-intensity exercise or physical activity intervention on patients with AD, we found that the concomitant effects on cognition functions of high frequency interventions was not greater than that of low frequency interventions, which is in consistency with what Groot C [52] et al. found that positive effect on cognition was smaller in high frequency interventions compared to low frequency interventions. Our study showed that interventions conducted up to 30 min per time have greater effectiveness for improving cognition of patients compared to those conducted more than 30 min per time; Interventions conducted up to 2 h per week have a slight tendency to show greater effectiveness for improving cognition of patients compared to those conducted more than 2 h per week; However due to the obvious heterogeneity between interventions conducted more than two hours a week and the limited number of interventions conducted up to 2 h a week, the evidence from the subgroup analysis of intervention hours per week is not strong enough to determine which subgroup is better. Interventions conducted less than 3 times including 3 times per week showed greater effect on improving cognition of patients compared to those conducted more than 3 times per week. Although Kramer et al [53], Van Gelder et al [54] did quantified studies finding that the minimal time of physical activity or exercise that could benefit cognition is 1.5 h per week for elderly. It is not clear if this could apply to patients with AD, we still need to find out the low limit time of single session that could exert positive effects on cognition of older adults with AD in that some of them are too weak to be physically active.

Although we could not draw a conclusion on whether high-intensity or moderate-intensity is better due to limited studies include, there is a noteworthy question need paying attention to that studies are not in consistent in the term of “intensity”. While some studies defined intensity in percentage of heart rate reserve [47, 55, 56], some did with maximal oxygen consumption (VO2peak) [57, 58] or percentage of maximal heart rate [59], a study even defined intensity as intervention minutes per session [60]. We deem it is urgent to set a standard system of definition involved in physical activity and exercise aimed at improving cognition of older adults with AD.

As for frequency, we think it is important to categorize the term frequency into the following three specific aspects (1). How long of intervention in a single session; (2). How long of intervention per week; (3) how many times intervention per week. In that results of our analysis show that of the above aspects may influence effects of exercise on cognitive function. But the best combination is yield to be discovered. By clarifying the term frequency, it is more likely to find a better intervention method to achieve goals in an accurate way.

In regard to the length of the whole duration, Ashwini K. Rao [60] concluded in a meta-analysis that longer duration of intervention was not associated with better outcomes. Rather, larger effect sizes were seen in studies with shorter and midrange intervention duration. While in our study, interventions conducted more than 16 weeks did have a better effect than those less than 16 weeks. But due to limited studies, the evidence is weak.

According to our results of this study, we would make a modest recommendation that patients with AD should do physical activity or exercise up to 3 times a week with 30 min per session. However this recommendation still needs verification by more targeted studies.

### Strengths, limitations, and future directions

The primary strength of our meta-analysis lies in its multiple-subgroup design, which provides better quantification of the associations between specified amounts of physical activity or exercise and relative effects on AD. Another strength is that only RCT studies were included. The included studies were conducted in both developed countries like Germany and Denmark and developing countries like China and Brazil, therefore these findings also apply to both kinds of countries. Last, we focused on a single type of dementia (AD), whereas past meta-analyses have typically included trials of subjects with multiple types of dementia [61–63] or subjects with a diagnosis only of cognitive impairment.

There are several limitations that might affect the reporting of our results. First, the review was limited to two languages English and Chinese. Hence, studies published in other languages were not captured, which may result in a limited number of include studies. Second, the types of physical activity or exercise are quite different, and the way researchers conducting interventions are not in identically standard criteria, so there is heterogeneity between included studies. Third, limited studies led to the failure to identify the optimal type to improve cognition of AD patients. And we failed to conduct the intensity analysis due to the restriction of data. Fifth, we cannot conclude on how long the effects will last in the reference group due to data restrictions. A majority of researchers seemed to have neglected this important issue, which warrants more attention in the future study that follow-up data is of vital importance. Sixth, we cannot exclude the potentially effects caused by some confounders. Due to the above limitations, more high-quality RCTS are needed to confirm the effects of physical activity and exercise on improving cognition of patients with AD in the future.

Standard criteria need to be established for future studies in several aspects. (1) Description of characteristics. Of all the reviewed RCT studies rendered ineligible, a majority (*n* = 22) we reviewed were deemed ineligible due to the lack of MMSE or scores not being presented in the way of Mean ± Standard Deviation; (2) Intervention design. Standard definition of terms as intensity, frequency, duration need to be settled to describe specific aspects of physical activity and exercise to find a more likely better intervention method to achieve goals in an accurate way. On the other hand, follow-up effects need to be in record to understand how long the effects could last. More RCTs with large sample are needed to verify the effects of physical activity and exercise on cognition of patients with AD around the whole world.

## Conclusion

Considering that there is no direct treatment for AD, it is urgent to find a way to delay its progress. Physical activity and exercise have been shown to aid in achieving this goal in a cost effective and sustainable manner. The results from this study shows that physical activity and exercise can achieve improvement in cognition of elderly with AD and high frequency interventions are not necessarily characterized with greater effects on cognition functions compared to low frequency interventions, we still need to find out the threshold of physical activity and exercise that could exert a positive effect on cognition performance of patients with AD. More high-quality randomized controlled trials with less methodological issues and noted heterogeneity are required to back up our present findings.

## Additional file

Additional file 1: PRISMA 2009 Checklist. (PDF 217 kb)

## Data Availability

All data generated or analysed in this study are included in this published article (and its Additional file 1).

## Abbreviations

AD: Alzheimer’s disease
CG: Control group
IG: Intervention group
MMSE: Mini-Mental State Examination
RCT: Randomized controlled trial
SD: Standard deviation
SMD: Standard Mean Difference
